# Endogenous Hyperandrogenism in Women and Its Influence on Exercise Capacity and Athletic Performance: A Narrative Review

**DOI:** 10.7759/cureus.110713

**Published:** 2026-06-12

**Authors:** Karolina Galas, Paulina Lewandowska

**Affiliations:** 1 Department of Internal Medicine, Medical University of Warsaw, Warsaw, POL

**Keywords:** differences of sex development, endogenous hyperandrogenism, exercise performance, female athletes, non-classic congenital adrenal hyperplasia, polycystic ovary syndrome, testosterone

## Abstract

The increasing importance of the issue of endogenous hyperandrogenism in women (which is found in polycystic ovary syndrome (PCOS), congenital adrenal hyperplasia (CAH), and differences of sex development (DSDs)) for both sports medicine and the regulation of female competitive athletics has made it necessary to examine whether high natural levels of androgens can improve exercise ability in women. It has been clearly demonstrated that exogenously administered androgens produce a physiologic benefit for athletes; however, little information exists regarding the physiologic benefits of endogenously elevated androgen levels. Androgens, specifically testosterone, could potentially impact several systems involved in physical activity, including bone formation and muscle hypertrophy, red blood cell production, and oxygen delivery to tissues, all of which would result in a potential physiologic advantage for certain types of athletes. Therefore, this review will discuss the existing literature examining the association between endogenously elevated androgen levels in women and their exercise capabilities (specifically muscle strength, aerobic capacity, hematologic parameters), while also evaluating the methodologic quality of the available literature. A comprehensive search of the medical literature using PubMed/MEDLINE and Google Scholar, along with manual reference screening, was conducted to identify relevant literature, including peer-reviewed articles. Current evidence suggests that excessive endogenous androgen levels are associated with greater amounts of lean body mass, higher hemoglobin concentrations, and better aerobic capacity compared to nonhyperandrogenemic women. The greatest amount of consensus was observed among women with DSDs. However, the data obtained from women with PCOS and CAH have been inconsistent. Thus, based upon the currently available evidence, there may be a physiologic advantage resulting from endogenous hyperandrogenism in select groups of women. However, the extent of the effect varies by condition. Additional research using appropriately designed studies will be needed to further define these relationships and provide direction for clinical practice decisions and regulatory policies.

## Introduction and background

The role of androgens in athletic performance has become a subject of intense scientific, ethical, and policy-related debate, particularly in the context of female athletes with naturally high testosterone levels. While exogenous anabolic androgenic steroid (AAS) use in sports is well-documented and banned due to its performance-enhancing effects, the implications of endogenous hyperandrogenism remain more complex and controversial [1,2]. This distinction is important because exogenous androgen administration represents pharmacological enhancement, whereas endogenous hyperandrogenism results from naturally occurring biological variation or endocrine disorders. In addition, physical exercise itself may influence circulating androgen concentrations and hypothalamic-pituitary-ovarian axis activity in women, which further complicates the interpretation of hormonal findings in female athletes [3,4]. Experimental and clinical observations consistently demonstrate that androgen exposure enhances skeletal muscle protein synthesis, accelerates post-exercise recovery, and increases strength-related performance parameters, explaining the longstanding relevance of anabolic steroids in competitive sport [5]. Increasing evidence suggests that elevated levels of endogenous testosterone, whether associated with polycystic ovary syndrome (PCOS), congenital adrenal hyperplasia (CAH), or differences of sex development (DSDs), may influence muscle mass, strength, erythropoiesis, and other performance-related physiological traits [6,7].

Available evidence suggests that hyperandrogenic conditions may be associated with selected performance-related traits, particularly in sports involving strength, power, oxygen transport, or high training tolerance [2,4,8,9]. However, unlike exogenous AAS doping, endogenous androgen excess arises from physiological or pathological processes and does not involve intentional rule-breaking. This introduces ethical challenges in determining eligibility and fairness in women's categories. The Court of Arbitration for Sport's rulings and regulatory actions by bodies such as the International Association of Athletics Federations (IAAF) and the International Olympic Committee (IOC) underscore the difficult balance between protecting fair competition and respecting athletes' rights and health [10].

Clinical studies on CAH and PCOS provide useful insight into how chronic endogenous androgen excess may relate to physical performance, insulin sensitivity, and metabolic risk [10,11]. While athletes with hyperandrogenism may experience physiological benefits, this endocrine state is also associated with long-term health consequences, including cardiovascular risk and insulin resistance, requiring thoughtful medical and regulatory responses. This review aims to synthesize findings from clinical endocrinology, sports medicine, and regulatory perspectives to clarify how endogenous androgens influence female athletic performance, the health implications involved, and the current state of policy and controversy regarding eligibility in competitive sports.

## Review

Search strategy and evidence selection

This review was conducted as a narrative synthesis of the literature addressing endogenous hyperandrogenism and its potential impact on exercise capacity, athletic performance, and health outcomes in females. A focused literature search was used to identify studies relevant to the clinical, physiological, and sports-related consequences of endogenous androgen excess. The synthesis focused on publications examining conditions associated with hyperandrogenism, particularly PCOS, CAH, and DSDs, as well as studies discussing the regulatory and ethical implications of hyperandrogenism in competitive sport. The review included original clinical or experimental research, observational studies, endocrinology and sports medicine publications, and selected policy or position papers published in English. Studies were considered relevant if they involved female participants or athletes with endogenous androgen excess; evaluated physiological, metabolic, or performance-related effects of endogenous androgens; and reported at least one outcome related to exercise capacity, muscle mass, strength, aerobic performance, body composition, insulin sensitivity, cardiovascular risk, or sport participation. Priority was given to publications that directly explored the relationship between endogenous androgen exposure and traits potentially relevant to athletic performance, including lean body mass, oxygen-carrying capacity, muscular function, and metabolic adaptations.

In this review, endogenous hyperandrogenism refers to androgen excess arising from internal physiological or pathological sources rather than from exogenous hormone use. Therefore, papers dealing only with anabolic-androgenic steroid use, pharmacological testosterone administration, male-only populations, or endocrine disorders unrelated to androgen excess were not included in the main discussion. Review articles, commentaries, conference abstracts without sufficient methodological detail, study protocols, and reports lacking outcomes relevant to exercise capacity, performance, or clinical consequences were not discussed in detail. In addition, publications in which androgen excess was only a minor background characteristic, without meaningful analysis of its physiological or functional implications, were not retained as core evidence.

Study selection was based on relevance to the objective of the review and contribution to understanding the mechanisms, manifestations, and implications of endogenous hyperandrogenism in physically active females and athletes. Particular emphasis was placed on studies allowing integration of endocrinological, functional, and sport-specific perspectives, so that the final synthesis could capture not only biological mechanisms, but also their potential practical and regulatory significance.

Information sources and search

The literature search for this review was conducted using major biomedical and health science databases, including PubMed/MEDLINE and Google Scholar, to identify publications relevant to endogenous hyperandrogenism and its relationship with exercise capacity, athletic performance, and associated health outcomes in females. Additional articles were identified through manual screening of reference lists from pertinent review papers, original studies, and policy-related publications in the fields of endocrinology, sports medicine, and female athlete health.

The search strategy was designed to capture studies addressing both the biological mechanisms and the functional implications of endogenous androgen excess. Search terms included combinations of keywords and Medical Subject Headings related to endogenous hyperandrogenism, female athletes, testosterone, PCOS, CAH, DSDs, exercise capacity, physical performance, muscle mass, aerobic capacity, and sports eligibility. Terms relating to regulatory and ethical aspects of female competition and hyperandrogenism were also included to ensure that policy-oriented literature relevant to the topic was captured.

Search results were screened on the basis of title and abstract, followed by full-text evaluation of potentially relevant records. The final selection of studies was guided by their relevance to the aim of the review, with preference given to articles providing direct clinical, physiological, or sport-specific data on the effects of endogenous androgen exposure in females. Where appropriate, seminal older publications were retained if they contributed important mechanistic or conceptual insight, whereas more recent publications were prioritized for clinical and policy-related aspects.

Pathophysiology of endogenous hyperandrogenism

Endogenous hyperandrogenism in females is a complex pathophysiological condition characterized by excessive production or increased biological activity of androgens, primarily testosterone, androstenedione, and dehydroepiandrosterone (DHEA), originating from either the ovaries or the adrenal glands. Although these hormones are present in women at substantially lower concentrations than in men, they play important roles in normal reproductive, metabolic, and musculoskeletal physiology. Androgens contribute to sexual development, follicular maturation, libido, bone metabolism, erythropoiesis, and the maintenance of lean body mass. Their physiological effects depend not only on total circulating concentrations but also on free hormone levels, sex hormone-binding globulin (SHBG) availability, peripheral conversion, and androgen receptor sensitivity. Dysregulation at any of these levels may lead to clinically relevant androgen excess, with manifestations ranging from menstrual dysfunction, acne, hirsutism, and virilization to broader systemic consequences involving insulin resistance, altered body composition, lipid abnormalities, and cardiovascular risk [12]. Thus, endogenous hyperandrogenism should be understood not merely as elevated serum androgen levels but as a spectrum of endocrine disturbances with reproductive, metabolic, and functional implications.

Hormonal regulation of androgens

Androgen production in females is regulated mainly by the hypothalamic-pituitary-gonadal and hypothalamic-pituitary-adrenal axes. Ovarian androgen synthesis occurs primarily in the theca cells under stimulation by luteinizing hormone (LH), leading to the production of androstenedione and testosterone. These androgens may then serve as substrates for estrogen synthesis in granulosa cells through aromatase activity, which is stimulated by follicle-stimulating hormone (FSH). Therefore, normal ovarian steroidogenesis depends on the coordinated interaction between LH-driven androgen production and FSH-dependent conversion of androgens into estrogens [13].

The adrenal contribution arises mainly from the zona reticularis of the adrenal cortex, where adrenocorticotropic hormone (ACTH) stimulates the production of dehydroepiandrosterone (DHEA), DHEA-sulfate (DHEAS), and androstenedione. DHEA and DHEAS have relatively weak intrinsic androgenic activity, but they function as important circulating precursors that can be converted in peripheral tissues into more potent androgens, including testosterone and dihydrotestosterone (DHT) [14]. The final biological effect of these hormones is influenced not only by their serum concentration but also by SHBG levels, peripheral enzymatic conversion, and androgen receptor sensitivity. Consequently, even moderate changes in ovarian or adrenal steroidogenesis may significantly alter free androgen availability and produce clinically relevant hyperandrogenic effects. Intensive physical training may itself alter hypothalamic-pituitary-ovarian axis regulation through chronic metabolic and neuroendocrine stress, potentially modifying gonadotropin secretion and ovarian steroidogenesis in female athletes [15].

Mechanisms of androgen excess

Hyperandrogenism develops when androgen production exceeds normal physiological levels or when the bioavailability of circulating androgens becomes abnormally increased. This may result from excessive ovarian or adrenal androgen synthesis, altered steroid metabolism, or reduced clearance of circulating hormones. Mechanistically, androgen excess may occur due to enhanced LH stimulation of ovarian theca cells, elevated adrenocorticotropic hormone (ACTH) secretion, or enzymatic abnormalities affecting steroidogenesis, such as 21-hydroxylase deficiency in CAH [13,14]. These disturbances increase the production of androgen precursors, including androstenedione and dehydroepiandrosterone (DHEA), which can subsequently be converted into testosterone and dihydrotestosterone (DHT) in peripheral tissues.

In many patients, insulin resistance plays an important permissive role in the development and maintenance of hyperandrogenism. Hyperinsulinemia suppresses hepatic production of SHBG, resulting in increased concentrations of biologically active free testosterone [16,17]. In addition, insulin can directly stimulate ovarian androgen synthesis and potentiate the effects of LH on theca cells, further amplifying androgen production. This interaction creates a self-reinforcing cycle in which androgen excess promotes abdominal adiposity and metabolic dysfunction, while worsening insulin resistance further increases androgen availability.

The clinical manifestations of hyperandrogenism are also influenced by peripheral androgen metabolism and tissue sensitivity to androgens. Weak adrenal androgens, such as DHEA and DHEAS, may undergo conversion in peripheral tissues into more potent androgens, particularly testosterone and DHT. Therefore, the severity of hyperandrogenic symptoms depends not only on total serum androgen levels but also on SHBG concentration, peripheral enzymatic activity, and androgen receptor sensitivity [14].

In PCOS, these mechanisms converge through altered GnRH/LH signaling, relatively impaired FSH-dependent follicular maturation and aromatization, hyperinsulinemia-related reduction in SHBG, and additional genetic, inflammatory, and oxidative-stress-related contributors [13,14,18-21]. Therefore, PCOS-related hyperandrogenism represents a combined endocrine and metabolic disturbance rather than a single isolated abnormality of androgen production.

Ovarian vs. adrenal sources

Differentiating between ovarian and adrenal sources of androgen excess is clinically important, as the underlying etiology influences both diagnostic evaluation and treatment strategies. Ovarian hyperandrogenism is most commonly associated with PCOS, which typically presents with menstrual irregularities, chronic anovulation, hirsutism, acne, and polycystic ovarian morphology on ultrasound examination [14]. In these patients, androgen excess is usually driven by increased LH-mediated stimulation of ovarian theca cells and is frequently accompanied by insulin resistance and metabolic dysfunction.

Adrenal causes of hyperandrogenism include non-classic congenital adrenal hyperplasia (NCCAH), adrenal hyperplasia, and androgen-secreting adrenal tumors. These conditions are more strongly associated with elevated levels of adrenal androgens, particularly dehydroepiandrosterone sulfate (DHEAS), and may present with more severe or rapidly progressive virilization [14,22]. Clinical findings such as deepening of the voice, clitoromegaly, rapidly developing hirsutism, or significant muscle hypertrophy should raise suspicion for a pathological adrenal source. Rarely, adrenal or ovarian androgen-secreting tumors may also cause severe hyperandrogenism and should be considered when androgen levels are markedly elevated or symptoms progress rapidly, particularly in the presence of virilization [22].

Insulin resistance and LH/FSH imbalance

Insulin resistance is a major contributor to the development and maintenance of hyperandrogenism, particularly in women with PCOS. Hyperinsulinemia increases androgen bioavailability through suppression of hepatic SHBG production, resulting in higher circulating levels of free testosterone [17]. In addition to this indirect effect, insulin can directly stimulate ovarian theca cells and enhance steroidogenesis, leading to increased production of androstenedione and testosterone. Insulin also acts synergistically with LH, further amplifying ovarian androgen synthesis.

Hyperandrogenic states are frequently associated with disturbances in gonadotropin secretion, characterized by elevated LH concentrations and relatively low or normal FSH levels [13]. This altered LH/FSH ratio promotes excessive androgen production by ovarian theca cells while impairing normal follicular maturation and ovulation. Reduced FSH stimulation may additionally limit aromatase activity in granulosa cells, decreasing the conversion of androgens into estrogens and contributing to further androgen accumulation. Together, insulin resistance and gonadotropin imbalance create a self-perpetuating endocrine environment that favors persistent hyperandrogenism and reproductive dysfunction.

PCOS

PCOS is the most common cause of hyperandrogenism in women, affecting up to 10% of females of reproductive age [14]. It is a heterogeneous endocrine and metabolic disorder characterized by varying combinations of hyperandrogenism, oligo- or anovulation, and polycystic ovarian morphology. Clinical manifestations commonly include hirsutism, acne, menstrual irregularities, infertility, and weight gain, although the severity and presentation may differ substantially between patients.

The pathophysiology of PCOS involves ovarian dysfunction, insulin resistance, and neuroendocrine abnormalities. Increased LH-mediated stimulation of ovarian theca cells, relatively insufficient FSH-dependent aromatization, hyperinsulinemia, and reduced SHBG collectively increase free androgen availability and contribute to anovulation and reproductive dysfunction [13,17]. Beyond reproductive effects, PCOS is associated with impaired glucose tolerance, dyslipidemia, metabolic syndrome, and increased long-term cardiovascular risk [16,17,21,23].

Exercise is clinically relevant in PCOS because it may influence several mechanisms involved in the syndrome, including insulin resistance, abdominal adiposity, inflammatory activity, oxidative stress, and hypothalamic-pituitary-ovarian axis regulation. Evidence from exercise intervention studies suggests that improvements in cardiorespiratory fitness and insulin sensitivity may occur even when changes in total body weight are limited. Therefore, in the context of endogenous hyperandrogenism, PCOS should be considered not only as a reproductive and metabolic disorder but also as a condition in which hormonal status may interact with exercise adaptation and functional capacity [20,21,24].

NCCAH

NCCAH is a milder and late-presenting form of 21-hydroxylase deficiency that results in adrenal hyperandrogenism due to impaired cortisol synthesis [14]. Reduced cortisol production disrupts normal negative feedback within the hypothalamic-pituitary-adrenal axis, leading to chronic elevation of adrenocorticotropic hormone (ACTH) secretion. Persistent ACTH stimulation promotes adrenal hyperplasia and increased production of adrenal androgens, including dehydroepiandrosterone (DHEA), androstenedione, and testosterone.

Compared with other forms of hyperandrogenism, NCCAH has been less frequently studied as a clinical model for assessing the effects of endogenously elevated androgens on physical performance. However, because it is characterized by chronic adrenal androgen excess, it may be useful for this purpose. Theoretically, excess adrenal androgen secretion may influence aerobic capacity, muscle strength, lean body mass, and hemoglobin concentration. Nevertheless, very few studies have specifically evaluated performance-related outcomes in females with NCCAH [11,14]. Because symptoms such as hirsutism, acne, irregular menses, and fertility problems may overlap between NCCAH and PCOS, basal or ACTH-stimulated 17-hydroxyprogesterone testing remains important for distinguishing these conditions [14].

DSDs

DSDs are a diverse group of congenital conditions affecting chromosomal, gonadal, or anatomical sex development. They are relevant to the discussion of endogenous hyperandrogenism in sport because some DSD conditions may involve naturally high testosterone levels or increased androgen exposure during development. In individuals with preserved androgen sensitivity, this may influence traits related to performance, including muscle mass, hemoglobin concentration, oxygen transport, bone structure, and recovery after training [2,6,9].

However, DSD should not be discussed as one uniform condition. The physiological effects of androgens depend on the specific diagnosis, timing and duration of exposure, androgen receptor sensitivity, training background, and the demands of a given sport. For this reason, testosterone concentration alone cannot reliably predict athletic performance in every female athlete [9,25].

DSD-related hyperandrogenism has also become an important issue in eligibility regulations for female sport. These policies attempt to balance fair competition with athlete privacy, inclusion, and medical ethics, but testosterone-based thresholds remain controversial [9,10,26,27]. Overall, DSD provides a useful model for studying the effects of endogenous androgens on performance, but direct evidence remains limited.

Effects of androgens on body composition and performance

Androgens have biologically plausible effects on skeletal muscle, erythropoiesis, body composition, and exercise-related adaptation [1-3,6,8,9]. Testosterone can act through androgen receptors in skeletal muscle and may contribute to muscle protein synthesis, lean body mass maintenance, and recovery after training stimuli [1,3,28]. Androgens may also influence erythropoiesis, hemoglobin concentration, and oxygen transport capacity, which are relevant to aerobic performance [2,6,8,9]. These mechanisms are well established in the context of exogenous anabolic-androgenic steroid exposure, but their magnitude and consistency in women with endogenous hyperandrogenism remain less certain [1,25].

The relationship between endogenous testosterone and physical performance in women is not linear and should not be interpreted as a simple dose-response effect across all populations [25]. A systematic review of observational studies did not find a consistent association between total testosterone and muscle mass, strength, or performance in community-dwelling women [25]. Some associations were reported between calculated free or bioavailable testosterone and lean mass, but these findings require cautious interpretation because of methodological limitations, including hormone assay variability and the use of calculated androgen indices [25].

Clinical and athlete-based studies provide condition-specific examples rather than a uniform dose-response model. Evidence from PCOS intervention studies and athlete cohorts suggests possible associations between hyperandrogenism and selected traits such as lean body mass, strength adaptation, aerobic capacity, bone mineral density, or training tolerance, whereas systematic evidence in broader female populations remains inconsistent [2,4,6,8,25,29]. These findings are discussed more specifically in the PCOS-focused section below.

Beyond performance-related traits, chronic androgen excess may also influence adipose tissue distribution and cardiometabolic risk. Women with PCOS and hyperandrogenism more frequently demonstrate insulin resistance, central adiposity, dyslipidemia, endothelial dysfunction, and increased cardiovascular risk markers, which may coexist with or counterbalance potential performance-related advantages [16,17,21,23]. Therefore, the effects of endogenous hyperandrogenism should be interpreted as condition-specific and multifactorial, involving potential anabolic and erythropoietic effects, metabolic consequences, training adaptation, and individual biological variability [2,4,6,8,17,25].

Androgens may also exert adverse cardiovascular effects in females. Experimental and clinical studies suggest that excessive testosterone exposure can contribute to endothelial dysfunction, increased arterial stiffness, elevated blood pressure, and unfavorable lipid profile alterations [23]. Similar findings have been reported both in women with PCOS and in transgender men receiving testosterone therapy. These observations support the concept that androgens exert sex-specific vascular effects and may predispose hyperandrogenic women to long-term cardiovascular complications despite potential improvements in physical performance and body composition.

PCOS, exercise adaptation, and athletic performance

PCOS represents one of the most clinically relevant models for evaluating how endogenous hyperandrogenism interacts with metabolic status, exercise adaptation, and physical performance in women [6,17,21]. Its interpretation is difficult because potential androgen-related advantages may coexist with insulin resistance, adiposity, and cardiovascular risk, which can negatively affect health and exercise tolerance [4,21].

Exercise interventions in women with PCOS appear to improve several physiological outcomes relevant to both health and physical capacity. Systematic evidence suggests that training, particularly vigorous-intensity aerobic exercise, may improve cardiorespiratory fitness, waist circumference, and insulin resistance in women with PCOS [24]. Resistance training may also be relevant, as progressive resistance training has been associated with increased muscle strength, increased lean body mass, and reduced body fat in women with PCOS [30]. These findings suggest that exercise may modify not only metabolic risk but also functional capacity and training responsiveness in hyperandrogenic women [24,30].

From a sports-performance perspective, the available evidence remains suggestive but not conclusive. Studies of female athletes with menstrual disturbances have identified hyperandrogenic subgroups with higher androgen levels, lower SHBG, more anabolic body composition, higher bone mineral density, and higher VO2max compared with normoandrogenic athletes or sedentary controls [29]. Similar reviews suggest that hyperandrogenism and PCOS may be more frequent in some athlete populations and may be relevant to selected performance-related traits, although causality remains uncertain [4]. However, data from broader female populations do not support a simple direct association between total testosterone and muscle mass, strength, or performance [25]. Therefore, PCOS-related hyperandrogenism should be interpreted as a possible modifier of selected physiological traits and training adaptation rather than as a universal determinant of athletic success [4,25,29].

Discussion

The available evidence supports a cautious interpretation: endogenous hyperandrogenism may modify selected performance-related traits through effects on skeletal muscle, erythropoiesis, hemoglobin concentration, oxygen transport, recovery, and body composition, but it does not establish a uniform relationship between serum testosterone and athletic performance in women [1,2,4,6,8,9,25,31]. Observations in selected athlete cohorts and hyperandrogenic conditions suggest associations with more anabolic body composition, higher bone mineral density, higher VO2max, explosive performance, or training tolerance in specific contexts [4,6,8,29]. However, athletic performance remains multifactorial and depends on training history, genetics, nutrition, body composition, androgen receptor sensitivity, sport discipline, and hormone-measurement methodology [2,6,8,25,31].

The syndrome illustrates this complexity particularly well. On one hand, PCOS-related hyperandrogenism may be associated with greater lean mass, strength adaptation, or overrepresentation in some athletic populations [4,6,8,30]. In affected women, progressive resistance training has been associated with improved muscle strength, increased lean body mass, reduced body fat, and decreased testosterone concentration after training [30]. More broadly, exercise interventions in PCOS appear to improve cardiorespiratory fitness, waist circumference, insulin sensitivity, and selected metabolic outcomes, especially when vigorous-intensity exercise is used [24]. On the other hand, PCOS is also associated with insulin resistance, abdominal adiposity, dyslipidemia, endothelial dysfunction, elevated blood pressure, and increased cardiometabolic risk, which may negatively affect long-term health and exercise tolerance [16,17,21,23]. Therefore, PCOS-related hyperandrogenism should not be interpreted only as a potential performance-enhancing condition but also as part of a broader endocrine and metabolic disorder requiring clinical consideration [13,14,17,21,24].

The interpretation of performance-related effects is further complicated by the fact that exercise itself may modify endocrine function in women. Physical training can influence circulating androgen concentrations, menstrual function, and hypothalamic-pituitary-ovarian axis activity, although the direction and magnitude of these changes depend on exercise type, intensity, energy availability, menstrual status, and hormonal measurement methods [3,4]. This means that androgen levels observed in athletes may reflect both pre-existing endocrine characteristics and adaptations to chronic training. Consequently, it is difficult to determine whether hyperandrogenism contributes to athletic selection, results from intensive training, or represents a combination of both mechanisms [4,29].

At the same time, endogenous hyperandrogenism differs fundamentally from exogenous anabolic-androgenic steroid use. Unlike intentional doping, endogenous androgen excess results from naturally occurring biological variation or pathological endocrine conditions [1,4,5]. This distinction creates major ethical and regulatory challenges regarding eligibility criteria in female sports [9,10,26]. Regulatory policies attempt to balance fairness in competition with inclusion, athlete autonomy, privacy, and medical ethics. Nevertheless, the scientific basis for fixed testosterone thresholds remains debated, particularly because athletic performance cannot be explained solely by serum testosterone concentrations [9,10,25,26].

An additional challenge lies in the heterogeneity of hyperandrogenic conditions. PCOS, NCCAH, and DSDs differ substantially in pathophysiology, androgen exposure duration, receptor sensitivity, metabolic consequences, and clinical presentation [13,14]. Consequently, the physiological impact of androgen excess likely varies considerably between individuals and conditions. Evidence from CAH suggests that androgen excess may influence exercise capacity, but these findings cannot be automatically generalized to PCOS or DSD because each condition involves different mechanisms, timing of this hormonal excess, and associated health consequences [11,13,14]. Available studies are also limited by small sample sizes in selected athlete cohorts, differences in study design, inconsistent hormone-measurement methods, variable control for training status and nutrition, and difficulty separating endocrine effects from genetic and environmental influences [4,25,29].

The long-term health implications of hyperandrogenism require careful consideration. Although elevated androgen exposure may be associated with selected performance-related traits, chronic hyperandrogenic states may also be linked to insulin resistance, central adiposity, dyslipidemia, endothelial dysfunction, hypertension, and increased cardiovascular risk [16,17,21,23]. Therefore, hyperandrogenic conditions should not be viewed solely through the lens of athletic performance enhancement but also as clinically relevant endocrine and metabolic disorders requiring appropriate diagnosis, follow-up, and individualized management [13,14,17,21].

Future research should focus on longitudinal studies evaluating objectively measured androgen concentrations, androgen receptor sensitivity, body composition, hemoglobin concentration, training adaptation, and sport-specific performance outcomes across different female populations. Particular attention should be given to distinguishing the effects of PCOS, CAH, and DSDs rather than treating endogenous hyperandrogenism as a single uniform condition [13,14,25]. Better-designed studies using precise hormone assays and standardized performance outcomes are needed to clarify the magnitude of androgen-related effects and to support evidence-based, ethically balanced approaches to female athlete health and eligibility regulation [9,10,25,26].

## Conclusions

Endogenous hyperandrogenism may influence exercise capacity in women through its effects on lean body mass, muscle strength, erythropoiesis, oxygen transport, and metabolic regulation. However, the available evidence remains heterogeneous and differs substantially between conditions such as PCOS, CAH, and DSDs. Although elevated endogenous androgen exposure may provide a physiological advantage in selected athletic contexts, its impact cannot be interpreted independently of training status, genetic background, androgen receptor sensitivity, and associated metabolic comorbidities. Current data are insufficient to define a universal relationship between testosterone concentrations and athletic performance in women. Further well-designed clinical and sport-specific studies are needed to clarify the magnitude of these effects and to support evidence-based, ethically balanced approaches to female athlete health and eligibility regulation.
